# Eye Behavior During Multiple Object Tracking and Multiple Identity Tracking

**DOI:** 10.3390/vision3030037

**Published:** 2019-07-31

**Authors:** Jukka Hyönä, Jie Li, Lauri Oksama

**Affiliations:** 1 Department of Psychology, University of Turku, FI-20014 Turku, Finland; 2 Institutes of Psychological Sciences, Hangzhou Normal University, Hangzhou 311121, China; 3 School of Psychology, Beijing Sport University, Beijing 100084, China; 4 Finnish Defence Research Agency, Human Performance Division, P.O. Box 5, FI-04401 Järvenpää, Finland

**Keywords:** eye movements, multiple object tracking, multiple identity tracking, dynamic attention

## Abstract

We review all published eye-tracking studies to date that have used eye movements to examine multiple object (MOT) or multiple identity tracking (MIT). In both tasks, observers dynamically track multiple moving objects. In MOT the objects are identical, whereas in MIT they have distinct identities. In MOT, observers prefer to fixate on blank space, which is often the center of gravity formed by the moving targets (centroid). In contrast, in MIT observers have a strong preference for the target-switching strategy, presumably to refresh and maintain identity-location bindings for the targets. To account for the qualitative differences between MOT and MIT, two mechanisms have been posited, a position tracking (MOT) and an identity tracking (MOT & MIT) mechanism. Eye-tracking studies of MOT have also demonstrated that observers execute rescue saccades toward targets in danger of becoming occluded or are about to change direction after a collision. Crowding attracts the eyes close to it in order to increase visual acuity for the crowded objects to prevent target loss. It is suggested that future studies should concentrate more on MIT, as MIT more closely resembles tracking in the real world.

## 1. Introduction

In many real-life visual tasks, people are required to keep track of multiple moving objects. This is true, for example, of car drivers maneuvering a vehicle across a busy intersection where the driver has to keep track of other vehicles approaching the intersection and of pedestrians crossing the street. This is also true of a football player making the decision of whom to pass the ball. When making that decision under stringent time constraints, he or she needs to be aware of both where his/her own teammates are as well as the players of the opponent team are located and moving toward. In the human factors’ literature, such awareness is called situation awareness [1]. It is genuinely dynamic in nature in that the visual environment (e.g., the intersection or the football field) is constantly changing, so that situation awareness has to be updated accordingly in order to adequately represent the moment-to-moment fluctuations in the relevant task environments.

**Figure 1:**
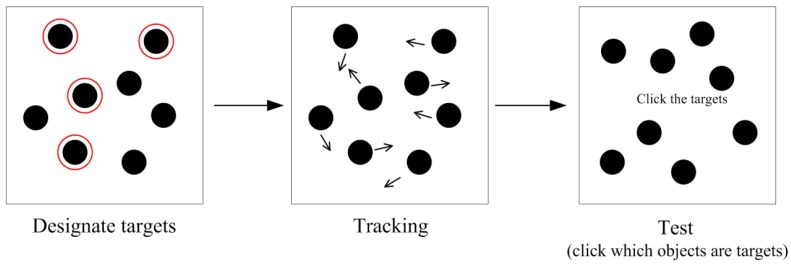
In the multiple object tracking (MOT) task, the targets to be tracked are first designated, for example, by drawing a red circle around the targets. After the target designation, the target and distracter items (all identical to each other) move for a few seconds. For the test phase, all objects stop moving and the participant is asked to click on the targets.

The experimental research on the dynamic tracking performance has mimicked the real-life visual environments using two types of laboratory tasks: multiple object tracking (MOT; for a review, see [2]) and multiple identity tracking (MIT; [3]). In both tasks, the moving objects are presented on a computer screen. The pioneering study, [4] introduced the MOT task. In MOT, the objects to be tracked are visually identical (e.g., black circles) so that they can be differentiated from each other only by their spatiotemporal properties. In the beginning of the task, a subset of the objects is designated as the targets, the remaining objects are distracters. The MOT task is depicted in Figure 1. The seminal work [4] demonstrated that people can track about four identical objects. Later it was shown [3] that there are significant individual differences in the MOT performance.

**Figure 2:**
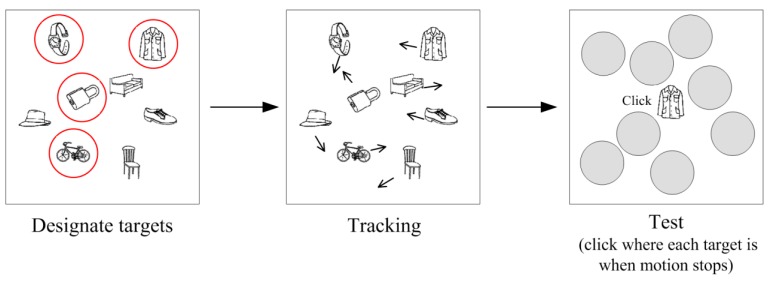
In the multiple identity tracking (MIT) task, all objects have distinct identities; in this example they are line drawings of common objects. In the initial target designation stage, the targets to be tracked are shown, for example, by drawing a red circle around them. Then all objects move for a few seconds. In the test phase, all objects stop moving and the targets are probed, for example, one by one by asking the participant to locate where each target was positioned at the time the movement was terminated.

The multiple identity tracking task was developed by Oksama and Hyönä [3,5]. It resembles more closely real-life visual environments in that the to-be-tracked objects all have distinct identities (like in traffic or sports). The MIT task is depicted in Figure 2. It was originally designed to mimic the visual environment air-traffic controllers or fighter pilots operate on. Air-traffic controllers monitor the incoming and outgoing aircraft depicted on a big screen by call signals that identify each aircraft. In the laboratory experiments the objects to be tracked have been, for example, line drawings of common objects or faces [5], cartoon characters [6] or words [7]. Horowitz et al. [6] demonstrated that tracking of multiple distinct identities is more difficult than tracking the whereabouts of identical objects. Perhaps not more than two objects with distinct identities can be successfully tracked.

The majority of MOT and MIT studies have examined the tracking performance by using performance accuracy as the dependent measure. However, there are some studies that have registered observers’ eye movements during the tracking performance. The eye-tracking studies of MOT and MIT are very useful, as they provide direct evidence regarding the allocation of overt attention during tracking. Thus, it may be used, for example, to study the extent to which observers overtly switch attention between the targets to perform the task. In this review paper, we go over all the eye-tracking studies conducted on MOT and MIT. We first review the MOT studied followed by the MIT studies and direct comparisons of the two tasks. We conclude the review by suggesting directions for future research.

## 2. Eye Behavior during MOT

### 2.1. Where Are Observers Looking at during MOT?

In this section, we review evidence on where observers look at during MOT. We first go over the studies providing evidence for the view that the center-looking strategy is the predominant strategy in MOT. Subsequently, we review evidence demonstrating that the target-looking strategy is in fact as dominant as the center-looking strategy. A summary of the eye movement studies of MOT is provided in Table 1.

#### 2.1.1. Center-Looking Strategy

##### The Central Areas between Targets are Frequently Gazed at during MOT 

An intriguing question is where observers are looking at when tracking multiple targets moving around in the visual field. In the seminal eye-tracking study of MOT, Fehd and Seiffert [8] provide evidence for the view that observers predominately look at the central area between the targets, presumably for grouping multiple targets into one single virtual object to track [21]. 

In the main experiment of the study, eye movements were registered when observers tracked 1 or 3 target dots out of 8 dots, which randomly moved about on the screen for 3 s with a speed of 15°/s. The trials in which the observers correctly tracked the targets were included in eye movement analyses. The 8 dots and the center of the targets (i.e., the centroid of the triangle formed by the 3 targets, which is the intersection of the medians) were defined for each frame as the competing areas of interest (AoIs). A location competition analysis with the shortest-distance rule was used to determine the location to which the gaze was closest. Each AoI was assigned a weight of zero at the beginning of the trial, and after each frame the AoI closest to the gaze position received an increase in its weight while the remaining AoIs received a decrease in weight. The AoI with the highest weight value on a given frame was considered the winner for that frame. The time each AoI was the winner across all frames was summed up, and then the average percentage of the time that the gaze was directed towards each AoI was calculated.

Their results showed that in the 1-target trials, the vast majority of the gazes (96.0%) were closest to the target dot. This is understandable as observers did not need to distribute their attention across multiple targets. More importantly, when tracking 3 targets, 65.7% of the time the gaze was closest to the center of the targets, while 8.5% of the gaze time was on each target dot, and only 1.0–2.2% on each distracter. The researchers replicated the finding in a follow-up experiment, which showed that participants looked significantly more at the center (41.6–42.4%) than at each of the targets when tracking 3 (10.7%), 4 (9.3%), and 5 (8.1%) targets in an array of 10 dots. Fehd and Seiffert [8] conclude that, “when multiple objects are tracked, more time is spent looking towards the center of the target array than at each target individually” (p. 206).

Such a center-looking strategy was replicated or partially replicated by subsequent eye-tracking MOT studies [9,10,13]. For example, Zelinsky and Neider [10] adopted an experimental setting somewhat different from the basic MOT paradigm, as they mimicked real-life dynamic visual environments by presenting on the screen moving sharks that moved underwater. Underwater scene is free of contextual constraints, as sharks can freely select and change their movement trajectory. In terms of features, sharks are more complex objects than dots, thus better approximating the kind of objects tracked in real life. In the experiment, 9 identical sharks moved about for 20 s in a 3D underwater scene (i.e., movement in depth was also rendered possible). Target set-size varied from 1 to 4. When the movement stopped, one of the sharks was probed by drawing a red circle around it, and the participant had to decide whether or not it was a target. The results showed that when tracking just one target, observers spent 94% of the time gazing at the single target; when tracking 2, 3, or 4 targets, observers spent 47%, 39%, 24% of the gaze time at the center.

##### Center-Looking Includes Centroid-Looking, Anticrowding, and More

Whereas, there is a consensus that observers frequently look at the central areas between multiple targets, it is more controversial which specific central locations observers are looking at during MOT and what strategies their eye behavior represents.

Fehd and Seiffert [8] propose that center-looking represents centroid-looking. Observers look at the centroid of the virtual polygon formed by the targets, which serves the function of mentally grouping multiple targets into one virtual object to track. In their analyses, they compared two types of central locations: the centroid of the virtual object for grouping, and the central points for minimizing the eccentricities of the targets (either the maximum eccentricity of any one target, or the average eccentricity of all the targets). The authors note that the eccentricity minimizing points are very close to the centroid, so it may be difficult to tell them apart, and hence they analyzed the frames where eccentricity minimizing points differed from the centroid by more than one degree. The result showed that the average distance to the gaze was larger from the eccentricity minimizing points than from the centroid (4.8–5.4° vs. 4.1°). Moreover, in the location competition analysis the eccentricity minimizing points were winners less often than the centroid (28.4–28.6% vs. 65.7%). Fehd and Seiffert [8] also compared the centroid with the central location calculated by averaging the coordinates of the targets. It is the same location when there are 3 targets but not when there are 4 or 5 targets. They did not describe explicitly how the centroid was defined when there were 4 or 5 targets. Presumably, it was defined as the geometric center of the polygon formed by the targets. Their results showed that the location of the average target coordinates did not correspond to the gaze position as closely as the centroid. Overall, Fehd and Seiffert [8] considered the centroid-looking strategy to fit best to the data and being a more parsimonious explanation, as it presumably reflects a grouping strategy whereby the targets are grouped to form a single virtual object. 

However, the dominant centroid-looking strategy may originate from the analysis methods used for determining to which AoI each gaze position belongs. Only the seminal studies using the shortest-distance rule found the majority of the gazes being closest to the centroid [8,10]. With this rule, gaze was defined as either looking at the centroid or one of the objects depending on which the distance was shorter. Thus, many gazes on central areas were categorized as looking at the centroid, even though they were fairly distant from it. For instance, as mentioned above, in the Fehd and Seiffert’s [8] study, the average distance between gaze and centroid was 4.1°. Subsequent studies used AoIs of different size to define whether gaze is at the centroid or on individual objects; they found that the centroid-looking strategy accounted for about 4–30% of the total gaze time [9,12,13,16,20], in comparison to 40–66% found in the seminal studies using the shortest-distance rule [8,10]. In particular, using AoIs of 3.4° in size, Oksama and Hyönä [20] found 7% of gaze time on the centroid, in contrast to 21% on individual targets, 4% on distracters, 4% on the screen center, and about half of the time (48%) on blank area that was neither the centroid nor the screen center, implying that center-looking entails more than merely centroid-looking.

Lukavský [16] compared five different types of central locations representing five types of center-looking strategies during MOT. Three of them were the centroid, the eccentricity minimizing point for the targets, and the screen center; the other two were the eccentricity minimizing point for all objects, and the anticrowding point, which minimizes the ratio between each target’s distance from the gaze point and the distance from every distracter. It is noteworthy that the computation of the last two central locations takes into account both the locations of the targets and the distracters. Lukavský [16] compared the adherence of the observed data to the five models of the center-looking strategy, and found highest adherence for the anticrowding point, closely followed by the target eccentricity minimizing point and the centroid, whereas the adherence of the all-object eccentricity minimizing point was lower, and that of the screen center was lowest. Moreover, observers spent a larger percentage of time gazing at the anticrowding point than the target eccentricity minimizing point or the centroid (12.2% vs. 9.0% vs. 7.7%). Therefore, Lukavský [16] suggests that an important function of the center-looking strategy is to mitigate the effect of crowding and thus reduce the danger of confusing targets with surrounding distracters. 

#### 2.1.2. Target-Looking

##### Individual Targets are Frequently Gazed at during MOT

Besides the central areas, the individual targets are frequently looked at during MOT as well, whereas the non-targets are seldom looked at. In fact, the total gaze time on all the targets is usually comparable to or larger than the gaze time on central areas [8,10,12,16]. For instance, even though Fehd and Seiffert [8] emphasized the center-looking strategy, the target-looking strategy also featured prominently in their data. While the center-looking strategy accounted for 41.6–42.4% of the gaze time, each of the targets was gazed at for 10.7%, 9.3%, and 8.1% of the time when tracking 3, 4, and 5 targets. It means that the target-looking accounted approximately for about 32.1%, 37.2%, and 40.5% (i.e., 10.7% × 3, 9.3% × 4, and 8.1% × 5) of the gaze time in total, which is comparable to the center-looking time. The Zelinsky and Neider [10] study also observed pronounced target-looking. When there were 2, 3, 4 targets, the time spent on targets accounted for 37%, 42%, 52%, which is comparable to or even greater than that spent on the center (47%, 39%, and 24%, for 2, 3, and 4 targets, respectively). 

The percentages of target-looking and center-looking both varied with the size of AoIs used in the eye-tracking MOT studies, yet it seems that target-looking is always comparable in frequency or even higher than centroid-looking. For instance, Huff, Papenmeier, Jahn and Hesse [12] used AoIs of 1.3° to 2.2° in size, which was of the same size as the objects. The results showed about 10% of the gaze time on the centroid, and about 5–10% on each of the 3 targets on average, resulting in about 15–30% spent on the targets in total. In Oksama and Hyönä’s [20] study, AoIs of 3.4° were used, while the size of the objects was around 2°. The results showed 7% of the gaze time on the centroid, and 21% on all the targets in total. The number of targets varied from 2 to 5, so that the average time on each target was around 4–10%. In Lukavský’s [16] study, the centroid was defined by an AoI of 1°, equal to the size of the moving objects, whereas the AoIs for objects were 2° (this is not explicitly stated in the paper but implied by “AOIs defined by targets and distracters are four times larger”). The results showed 7.7% of the gaze time on the centroid, and 3.1% of the gaze time on each of the 4 targets on average, resulting in about 12.6% on the targets in total. Fehd and Seiffert [9] used two different sizes of AoIs in their Experiment 2. The size of the objects was 0.06–0.3°. They first used AoIs of 0.6°. The results showed very small percentages of gaze overlap with the AoIs. Gaze overlapped with the centroid 4.0% of the time, and 0.9% and 0.3% with each of the 4 targets and 6 distracters, resulting in 3.6% on targets and 1.8% on distracters. They then increased the AoI size to 5°. Gaze overlapped with the centroid for 34.3%, and 7.8% and 3.1% for each of the targets and distracters, resulting in 31.2% and 18.6% of gaze time on the targets and distracters, respectively. The percentages were similar to those in their Experiment 1 with 5° AoIs, which were about 25% on the centroid, 40% on the targets, and 18% on the distracters. In sum, across the different studies, as the AoI size varied between 0.6–5°, the range of the average percentage of gaze time on each target was about 1–10%, resulting in the total target-looking time of 4–40%, while the centroid-looking was about 4–34%. 

It has been demonstrated that observers frequently switch their gaze back and forth between the center and the targets rather than switching from target to target. Fehd and Seiffert [9] showed that in each trial, there were 5.9 center-to-target switches on average, which was significantly more than target-to-target switches (1.9). The results were replicated by Vater, Kredel, and Hossner [13], who found 5.5 center–target switches per trial in comparison with 1.3 target–target switches. The Oksama and Hyönä [20] study provided support for another possible strategy, namely the look-at-one-target-strategy. The results showed that regardless the number of the targets that needed to be tracked (varied from 2 to 5), the number of targets gazed at during tracking remained constant, about 1½ targets. It implies that observers may choose one target to be overtly tracked by the eyes, while the other targets are tracked peripherally in a covert fashion. “Such a strategy seems both psychologically and computationally more plausible than the continuous computation of the centroid dynamically delineated by the moving targets, which appears computationally quite demanding” [20]. It should be noted, however, that in their study the look-at-one-target-strategy was less frequent than the look-at-blank-space strategy.

##### Gaze to Individual Targets Lags behind the Targets Rather Than Extrapolate Target Motion

Another interesting question about target-looking is that when observers look at targets, whether the eyes extrapolate target motion and land on a position where the target is expected to go to. Lukavský and Děchtěrenko [18] examined this question yet failed to find evidence in support of motion extrapolation in MOT. Instead, they observed that in all the tested conditions the gaze lagged behind the targets. In their study, the stimuli comprised 8 gray disks of 1° of visual angle in size that moved in a constant speed of 5°/s. Four targets among 4 distracters were tracked for 10 s. Importantly, they presented forward motion trajectories, repeated for 4 times, and backward motion trajectories that were the forward trajectories played back in reverse order. The logic is that if there is no motion prediction, gaze behavior would be highly similar between the forward and backward trajectories. In order to make this comparison possible, the eye movement data were reversed for the backward trajectories. The procedure also allowed determining the temporal lag or lead time of gaze behavior in relation to object motion. 

Using the logic described above, they [18] observed in Experiment 1 (20 participants) an average lag of 114 ms in gaze behavior with respect to object motion, with all participants demonstrating a lag (from 52 ms to 173 ms). In Experiment 2 (27 participants), they manipulated target set-size (2 or 4) to investigate whether track load affects motion extrapolation. It was found that the gaze lagged behind object motion a bit less (a non-significant difference) with low (2 targets) than high (4 targets) track load (93 ms vs. 108 ms). In other words, an easy tracking condition did not lead to a significantly shorter lag. Yet, it should be noted that object motion was not very predictable. In Experiment 3, the predictability of object motion was manipulated. In Experiment 3a (23 participants), the trajectories used in Experiment 2 (baseline) were pitted against more predictable trajectories, while in Experiment 3b (28 participants) the baseline condition was compared against a less predictable (i.e., more chaotic) condition. It was observed that more predictable trajectories did not reliably shorten the gaze lag (although there was a tendency in that direction), but the more chaotic condition reliably delayed the lag by 32 ms (102 ms vs. 134 ms). It is suggested that the 100-ms lag may reflect oculomotor limits. Generally speaking, the study [18] failed to find evidence in support of motion extrapolation in MOT, as in all tested conditions gaze lagged behind the targets.

#### 2.1.3. Summary: Both Center-Looking and Target-Looking during MOT

In sum, during MOT, observers frequently look at the central areas between targets. The central locations mainly include the centroid of the virtual polygon formed by the targets, the anticrowding point, and the eccentricity minimizing point. The individual targets are frequently looked at as well, while non-targets are seldom looked at. The total time of target-looking is comparable to center-looking. Observers frequently switch the gaze back and forth between the center and the targets, manifesting a center-target-switching strategy; yet they may also overtly follow one target by gazing at it, while tracking the other targets peripherally in a covert fashion. When looking at the targets, the gaze does not extrapolate their motion trajectories but instead lags behind. 

It is noteworthy that the studies differ in the way eye movement data were analyzed. In most studies (e.g., [8,10]) raw data (i.e., × and y coordinates) are assigned to AOIs (for an open-source automated tool for conducting such analyses, see [22]), while in some studies (e.g., [20]) the raw data are first parsed into fixations and saccades, after which fixations are assigned to the AOIs. The downside of the former analysis procedure is that also saccades are signed to AOIs despite the fact that no visual information processing is carried out during saccades due to saccadic suppression. This may not be considered a serious problem, as saccades are rapid and thus consume relatively little time. On the other hand, the downside of the latter analysis procedure is that it cannot differentiate smooth pursuit movements from fixations. Yet, in our data, we seldom observe smooth pursuits when multiple objects are to be tracked. The situation is dramatically different when only one target is tracked; observers are likely to track the single target with smooth pursuit eye movements (see [8,10]).

### 2.2. What Influences Eye Behavior during MOT?

#### 2.2.1. Number of Target Objects

To date, the results are inconsistent regarding how eye behavior during MOT varies as a function of the number of targets. Clearly, when tracking one single target, observers spend almost all the gaze time (about 95%) on the target [8,10]. When tracking more than one target, some studies show that as the number of targets increases, the total target-looking time increases while the average looking time on each target decreases. For instance, Zelinsky and Neider [10] showed that as the number of targets increased from 2, 3, to 4, the total target-looking time increased from 37%, 42%, to 52%, while the average looking time on each target dropped from 18%, 14%, to 13%, respectively. Fehd and Seiffert [8] found a similar trend; by showing that as the number of targets increased from 3, 4, to 5, the total target-looking time increased from 32.1%, 37.2%, to 40.5%, while the average looking time on each target dropped from 10.7%, 9.3%, to 8.1%, respectively. Zelinsky and Neider [10] suggest that the increase in total target-looking time is due to observers shifting from the centroid-looking strategy to the target-switching strategy when there are more targets to track. This argument was supported by the finding that as the number of targets increased from 2, 3, to 4, the centroid-looking time decreased from 47%, 39%, to 24%. Moreover, Zelinsky and Todor [11] found that the gaze-target distance was generally larger for tracking 2 rather than 3 or 4 targets, perhaps reflecting a more pronounced centroid-looking strategy in set-size 2. In contrast, the Fehd and Seiffert [8] study suggests that the centroid-looking strategy remains constant regardless of the number of targets; as the number of targets varied from 3 to 5, the centroid-looking time remained at 41.6–42.4%.

In contrast to the seminal eye-tracking studies of MOT, Oksama and Hyönä [20] found that as the number of targets increased from 2 to 5, almost all the measures of eye behavior remained constant, including the number of eye visits to targets, the number of visited targets, average fixation duration, the number of fixations, pupil size, and the number of blinks. The results provide strong support for parallel tracking models of MOT. The discrepancy between the studies may originate from the variation in motion speed, as discussed below. 

#### 2.2.2. Motion Speed

Fehd and Seiffert [9] argue that regardless of target speed, the prevalence of the centroid-looking strategy remains unchanged. They examined the possibility that observers adopt the centroid-looking strategy only when individual targets are moving too fast to be followed with the eyes. The authors varied the motion speed from slow to very fast (3, 6, 12, 18, or 24°/s); yet, they found no effect of speed in the percentage of gaze time on the centroid, targets, and distracters. Moreover, the gaze time on the centroid was always longer than that on each target and distracter regardless of speed. On the other hand, the dwell time decreased as the speed increased, showing that “At the slower speeds, participants viewed the center for prolonged periods of time, while at higher speeds they made quick glances to the center” (p. 5). Similarly, Vater et al. [13] found no effect of speed in the percentage of gaze time on the centroid, target, and distracter, when motion speed was varied from 6°/s, 9°/s, to 12°/s.

In contrast, Huff et al. [12] provided evidence for the view that the relative importance of centroid-looking increases with object speed. They manipulated object speed to be 2, 4, or 6°/s. The results showed that when the speed was slow (2°/s), the percentage of gaze time on the centroid was even lower than that on a target (about 7% vs. 10%). As the object speed increased, the centroid-looking strategy became more frequent while the target-looking preference decreased. Huff et al. [12] speculate that the centroid-looking preference reflects its benefits over the target-switching strategy. The centroid’s velocity and degree of movement is lower than those of targets, with the difference being particularly pronounced at higher speed. This presumably explains why the centroid-looking preference is increased with higher object speeds.

Oksama and Hyönä [20] suggests that as the motion speed increases from low to high, observers switch from a more serial tracking to a more parallel tracking, manifested in decreased fixations on individual targets and increased fixations on the central areas between targets. The authors varied the speed from 2.6°/s (slow), 6.3°/s (medium), to 10.7°/s (fast), while varying the number of targets from 2 to 5. The results showed that as the object speed increased, the participants spent less time looking at the targets and more on the center. Moreover, when the speed was slow, the number of fixations and target visits increased while the fixation duration tended to decrease as the number of targets increased. On the other hand, with a medium speed, the number of fixations and target visits as well as the fixation duration remained constant as the number of targets increased. Finally, with a high speed, the trend was that the number of fixations and target visits decreased and the fixation duration increased as a function of the number of targets. The results indicate that at slow speed observers adopt a more serial tracking, switching the eyes from one target to another, while as the speed increases they adopt a more parallel tracking strategy. 

The discrepancy in the results reviewed above may originate from the differences in the tested object speeds. Thus, perhaps Huff et al. [12] found increased centroid-looking associated with an increase in motion speed, because the speed in their study varied from low to medium (2, 4, and 6°/s), whereas Fehd and Seiffert [9] and Vater et al. [13] found constant centroid-looking as a function of motion speed, because the speed in these studies varied mostly from medium to high.

Research has also shown that as object speed increases, observers make fewer saccades, presumably to reduce the risks of losing targets during saccadic eye movements. When objects are moving slowly, even if some information is lost due to saccadic suppression, it can still be easily recovered after the saccade, whereas recovery may be impossible at high motion speed, since the locations of the objects may have significantly shifted position during a saccade [17].

In sum, observers frequently switch the gaze from target to target when the targets are moving slowly, showing a pattern of tracking the targets in a serial fashion, whereas they exhibit more center-looking at medium to high object speeds, demonstrating parallel tracking of targets.

#### 2.2.3. Stimulus Size

In principal, the decrease in stimulus size leads to reduced peripheral resolution of the targets, so that observers may fixate on the individual targets more often and hence the centroid-looking strategy may be abandoned. Fehd and Seiffert [9] examined this possibility in their Experiment 2. Squares were used as stimuli. They varied in size from 1 to 5 pixels on a side, subtending 0.06° to 0.3° of visual angle, so that the largest stimulus was seven times smaller than the stimuli used in their Experiment 1. When using AoIs of the same size as in Experiment 1 (5° in diameter), the results revealed the same centroid-looking strategy as in Experiment 1, manifested in 34.3% of the gaze time on the centroid and 7.8% on each separate target. When using AoIs double the size of the stimuli, which were significantly smaller than in Experiment 1, the centroid was still gazed at more than each individual target, although the percentages were much smaller than in Experiment 1, 4.0% on the centroid and 0.9% on the targets. Furthermore, as size decreased from 0.3° to 0.12°, the percentage of gaze time on the centroid decreased from 5.0% to 3.2%, while the gaze time on targets increased from 0.9% to 1.3%, suggesting a small trend for decreased centroid-looking and increased target-looking with decreased stimulus size. Overall, the study showed that target-looking increases and centroid-looking still prevails when stimulus size is small, demonstrating that the centroid-looking strategy “provides some value to tracking objects beyond the use of peripheral vision” (p. 7).

#### 2.2.4. Crowding and Collision

During MOT, crowding and collision commonly co-occur and may influence the tracking performance and eye behavior. Crowding is established when the objects move close to one another so that it is more difficult to differentiate the targets from the distracters. At the same time, the probability for object collisions is increased, which may also impact tracking. Vater, Kredel, and Hossner [15] disentangled the effects of crowding and collision on eye movements during tracking. Collision was manipulated by having the targets collide with the bordering frame in one condition during a critical period of 0.5 s, which was compared to a no collision condition. Crowding was also manipulated by two conditions. In the crowding condition, during the critical period three of the four targets formed a group where each target was closely surrounded by a distracter (the fourth target was not); in the no-crowding condition, the group of three targets was not closely surrounded by distracters. 

The authors measured the distance between the gaze and the targets, as well as the number of saccades initiated to the targets in different conditions. Saccades to the targets were initiated much more frequently prior to the collision (64%) than after the collision (9%). These anticipatory saccades are assumed to be made to update changes in motion trajectories after the collision against the boundary. On the other hand, crowding did not affect the probability of initiating a saccade; yet, the gaze was located closer to the target group than to the isolated target in the crowding than in the no-crowding condition. The result is consistent with the study of [11], who found that the gaze-target distance decreased as the target-other-object distance became smaller. Similarly, the modelling studies of Lukavský and colleagues [16,17] observed that the anticrowding model is the best model for explaining the gaze locations during tracking. This is presumably due to attempts to increase visual acuity for targets in order to be better able to keep them separate from the distracters. In sum, crowding draws the eyes toward the crowded area, while collision induces anticipatory saccades to the target.

#### 2.2.5. Occlusion

Zelinsky and Todor [11] examined eye movements in a MOT task where objects occlude each other. The experiment was otherwise identical to their earlier study [10] except that in half of the trials at least one target, usually more than one, was involved in an occlusion. Observers tracked 2, 3 or 4 targets out of 9 objects for a period of 20 s. The main goal of the study was to assess the premise that observers “look at targets in order to prevent occlusion-related track losses” [11] (p. 4). In order to do that the authors examined the number of saccades programmed to occluded targets during the occlusion period (800 ms around the occlusion). These saccades were named as rescue saccades, as they are presumably carried out to prevent from losing track of occluded targets. 

The results showed that rescue saccades occurred in 45% of the occlusion periods, but only in 8% of the time before an occlusion period of the same duration. The rescue saccades were anticipatory in nature, as they frequently (33.7%) appeared within the time bin of 800 ms prior to occlusion. Interestingly, the probability of rescue saccades was at the same level (42.3%) in near-occlusion events where a target came close to another object (closer than 1° distance) and then moved away without being occluded, suggesting that close distance may signal forthcoming occlusion and hence triggers anticipatory saccades. In addition, the authors observed no difference in the frequency of rescue saccades between the target-target occlusions (or near-occlusions) and the target-distracter occlusions. This suggests that the process is driven by the possibility of confusion regardless of whether a target would be confused with a distracter or another target. The finding that the incidence of rescue saccades was no more likely with distracters than with targets makes sense by considering that in real-life tracking tasks, unlike in MOT, it is equally detrimental to confuse a target with another target as with a distracter. This is because in real-life tracking tasks the targets have distinct identities that have to be kept separate from each other. Thus, the predisposition of the eye movement system may be to automatically initiate saccades to targets in proximity to other objects. In sum, similarly to collisions, target occlusion induces anticipatory saccades to the targets.

#### 2.2.6. Abrupt Viewpoint Changes

Huff et al. [12] examined the effects of abrupt viewpoint changes in MOT on observers’ eye movement patterns. Changing abruptly the viewpoint resembles a situation where a person watches a football match on TV where the game is presented as sequential shots from different camera angles. In Experiment 1, eight white spheres (a size of 1.3 to 2.2°) moved on a floor plane resembling a checkerboard. Three spheres were designated as the targets. Objects moved for 5 s in straight lines in randomly chosen directions with a constant speed of 2, 4 or 6°/s. In half of the trials, after 3 s the camera viewpoint changed abruptly by 20° either to the left or right. At the end of the trial when the objects stopped moving, the participant had to click on the targets with a computer mouse. In Experiment 2, the authors [12] made three changes to the procedure. First, the timing of the abrupt viewpoint change was varied in order to prevent anticipation. Second, object speed was increased to 10°/s; this speed was compared to the medium speed condition of Experiment 1 (4°/s). Finally, participants were asked to report after each trial whether or not they were able to track all the targets.

The analyses showed that after the abrupt viewpoint change, there was a drop-in gaze time on targets. The drop was temporary, as after 500 ms following the viewpoint change, gaze time on targets returned to the level it was prior to the change. The drop-in gaze time was smaller and non-significant for centroid looking. The first saccade after the change was executed faster to the centroid than to a target. It suggests that a new centroid was readily recalculated. In the 500-ms time bin after the viewpoint change, there were also more saccades to the centroid than to the target. Moreover, these effects of viewpoint change on gaze behavior existed only when the motion speed was relatively slow (2–6°/s) but disappeared when the speed was fast (10°/s). Huff et al. [12] speculate that the centroid-looking preference reflects its benefits over the target-switching strategy. The viewpoint change affects less the location of the centroid than the targets, which is assumed to explain why the gaze on the centroid was less influenced by viewpoint change. Moreover, the centroid’s velocity and degree of movement is lower than those of targets. This difference is particularly pronounced with higher speeds. This presumably explains why the centroid-looking preference is increased with higher objects speeds. 

In sum, abrupt viewpoint changes do not substantially affect centroid-looking, as saccades are quickly made to the centroid after the change; meanwhile, target-looking temporarily drops after the viewpoint change. 

#### 2.2.7. Trajectory Repetition and Flipping 

Lukavský [16] investigated whether the repetition of motion trajectory would increase similarity in eye behavior. The repetition effects were studied by presenting the odd-numbered trials in the MOT experiments as unique trials, while the even-numbered trials reappeared once in each of the 4 blocks. Similarity in scanpaths was analyzed with a measure of normalized scan path saliency [23]. It was found that repeated trials produced more similar scanpaths than unique trials. This finding was replicated by using mean gaze distance between frames as the dependent measure. Observers were to some extent aware of the fact that some trials were repeated. In a post-experiment recognition test they were presented a subset of the trials and asked to judge whether or not they had seen them during the experiment. Repeated trials were recognized somewhat better than unique trials (47% vs. 39%). 

To prevent the contamination of observers noticing the repetition and consciously learning the trials, Děchtěrenko, Lukavský, and Holmqvist [19] geometrically flipped the trajectories along the *x*- or *y*-axes in the repeated trials. In addition, the trajectories shared common segments for 6 s while differed for the rest (2 s) of the motion. Using the measure of the correlation distance metric, the authors showed that even though the scan patterns in the flipped trials differed significantly from those in the original trials, the difference was small (as little as a 13% increase of overall distance). In sum, eye behavior generally remains constant when the trajectories of object motion are repeated or flipped.

### 2.3. What Are the Functions of Eye Behavior during MOT? 

In this section, we outline possible functions of eye movements carried out during MOT. The currently available research suggests that they may serve the function of grouping targets together, counteracting detrimental effects of crowding, occlusion and collision, as well as detecting changes in targets. 

#### 2.3.1. Centroid-Looking for Grouping

The idea put forth in the seminal eye movement studies of MOT was that the main function of eye behavior during MOT is to help group multiple targets as a single virtual polygon by gazing at the centroid of the polygon [9,10,21]. This idea was supported by the studies investigating the relationship between eye behavior and tracking performance. Zelinsky and Neider [10] computed correlations between the adopted eye movement strategy and tracking accuracy. The correlations demonstrated that increased time looking at the centroid was associated with better performance, whereas increasing time to look at the targets led to poorer performance.

Fehd and Seiffert [9] instructed the participants of their Experiment 3 to track the targets with three types of looking strategy: free-looking, center–looking, and target-looking. In the first experimental block the participants were free to use any strategy they wished, after which they were instructed to use the latter two strategies in different blocks. The center-looking instructions emphasized that the participants should “keep their gaze near the center point of the target group or near a target”, and “when they looked at one of the targets, to look back at the center before looking at another target.” The target-looking instructions emphasized that the participants should “keep their gaze near a target”, and “when they looked away from one target, to be sure to look at another target.” The results showed that the free-looking strategy resulted in better tracking accuracy (83%) than the center–looking (77%) or the target-looking (57%) strategy, while the center–looking strategy was reliably better than the target-looking strategy. Moreover, most of the participants preferred the center–looking strategy to the target-looking strategy, as during the free-looking block the center–looking strategy was used more frequently than the target-looking strategy (40% vs. 13%). It is concluded that “instructed center-looking is beneficial to tracking but not as helpful as allowing participants to move their eyes naturally” [9] (p. 9).

#### 2.3.2. Target-Looking and Center-Looking for Resolving Crowding

Target-looking is helpful for extracting high-resolution information of the targets in order to enhance the distinction between the targets and the distracters in crowding situations. Zelinsky and Todor [11] reported that people tend to look closer to the targets that are in the proximity of other objects. In their analysis, the gaze-to-target distance was plotted as a function of target-to-other-object (the other object could be a distracter or another target) distance. It was found that the gaze-target distance decreased as the target-other-object distance became smaller. For target set-size 2 and 3 this occurred primarily for short target-object distances, whereas for target set-size 4 the decline was linear. Moreover, the gaze-target distance was generally larger for set-size 2 than set-size 3 or 4. The results indicate that observers gaze closer to individual targets to resolve local crowding clusters. Similarly, Vater et al. [15] showed that “gaze is located closer to targets when they are crowded, as would be expected to reduce negative crowding effects by utilizing the higher spatial acuity of foveal vision” (p. 1).

On the other hand, one important function of center-looking is to gaze at the anticrowding point between the targets, which minimizes the ratio between each target’s distance from the gaze point and distance from every distracter, so as to reduce crowding globally. Such an anticrowding model shows highest adherence to the observed eye movement data, which even outperformed the model of centroid-looking [16,17]. 

#### 2.3.3. Detecting Changes in Object Form and Motion by Using Peripheral Vision

Vater et al. [13] investigated in Experiment 2 the detection of form and motion changes in peripheral vision during MOT. Motion change was operationalized as a sudden stop of target movement for 0.5 s, while form change was operationalized as a target changing for 0.5 s from square to diamond (square rotated by 45°). Assuming that the participants looked at the centroid at the time of the change (a specific manipulation was arranged to create a static centroid for the critical period), the change took place 15° into the periphery from the fixation point. The participants were asked to press a button as soon as they detected a change; at the end of the trial, a number was projected to each object, and the participants were required to name the number of the changed target. If no change occurred, they were to recall all four targets. Eye behavior was analyzed for trials where the target was correctly detected and the gaze was farther than 5° away from the to-be-changed target at the time the change was initiated. The results demonstrated significantly better detection of motion than form changes. Participants were also faster to do so, as indicated by the higher percentage in the motion than form change condition of button presses prior to fixating the changed target. On the other hand, a saccade to the changed target was initiated faster in the form than motion change condition, indicating that observers are more inclined to use foveal vision to inspect form than motion change. The average saccadic reaction times were above 0.5 s, which means that typically the saccade reached the target after the termination of the momentary (0.5 s) change. In other words, the changes were detected with the help of peripheral vision. The trials during which the critical saccade reached the target before the change was terminated did not result in better change detection than the trials where the saccade reached the target after the change had ended. This is taken as further proof that detection was achieved by peripheral vision. 

As a direct follow-up, Vater, Kredel and Hossner [14] manipulated the peripheral location of the change so that it took place either relatively near (5–10°) or farther away (15–20°) from the centroid. The results of Experiment 1 showed that eccentricity influenced the change detection success; changes were detected more poorly in the far than near eccentricities. Eccentricity also affected the detection speed for form changes but not for motion changes; form changes were slower to detect at far eccentricities. In Experiment 2 [15], two types of temporary changes in target motion needed to be detected: a temporary stop (similarly to Experiment 1) and a slowdown of motion. Motion changes were detected better and faster at near than far eccentricities. Moreover, change detection was faster in the stop than in the slowdown condition. In sum, it is concluded (see also [13]) that “peripheral vision is more capable of detecting motion than form changes due to its high motion sensitivity and low acuity” [14] (p. 912).

#### 2.3.4. Saccades Induced by Changes and Forthcoming Occlusion and Collision 

As mentioned earlier, Zelinsky and Todor [11] found that rescue saccades are made to targets that are about to be occluded. Furthermore, these saccades are anticipatory in nature in that saccades are made before the occlusion. The results demonstrate the instrumental nature of eye movements during MOT in the form of rescue saccades that are a crucial tool in selectively processing “targets that are in danger of being lost” due to occlusion [11] (p. 10). Yet, it is not known from this study whether tracking accuracy was actually improved by the execution of rescue saccades. 

Similarly, Vater et al. [15] showed that anticipatory saccades are made to targets that are about to collide with the border frame. In their study, a target-stop-detection task was adopted as the secondary task. The results showed that a change in target motion was detected poorer in the collision than the no-collision condition. The novel finding relevant to the present section is that it was confined to cases where an anticipatory saccade was executed toward the to-be-colliding targets. This suggests that saccades disrupt the continuous flow of visual information and thus impair change detection. Moreover, as mentioned in the previous section, a saccade could also be elicited by target changes. Typically, the saccade reached the target after the termination of the momentary change, and the saccade did not benefit change detection even in cases when it reached the target before the change had ended [13].

In sum, the results reviewed in this section showed that during tracking saccades are initiated to changed targets and targets that are about to be occluded or collided to each other. With good reasons, these saccades can be assumed to be beneficial to tracking, as indicated by their name (rescue saccades). Yet they may disrupt information processing due to saccadic suppression. Such eye behavior is likely to be automatically induced by lower-level visual processing, despite not necessarily being useful or being even detrimental to the tracking performance. Clearly, more research needs to be conducted on this issue before firmer conclusions can be made on its role in MOT.

### 2.4. Summary of MOT Studies: A Processing Architecture for Modeling Eye Movements during MOT

In sum, during MOT eyes frequently gaze at the central area between targets for grouping and anticrowding. Moreover, the eyes are located closer to crowded targets to extract high-resolution location information. High motion speed and abrupt viewpoint change lead to more gazes performed towards the center of the targets, presumably for anchoring the reference frame and tracking the targets in parallel. Repeated or flipped trajectories result in more similar eye behavior than unique trajectories, whereas the eye gaze at the targets does not extrapolate the target trajectories but lags behind the targets. Changes in target form and motion, as well as forthcoming occlusion and collision, are perceived in the peripheral vision and induce saccadic eye movements towards the relevant targets. Even though they may be assumed to benefit tracking, they may actually disrupt object tracking due to saccadic suppression.

We suggest that eye movements during MOT should be understood in relation to the processes of object tracking, which contain different levels of processing. We propose that for successful tracking, the process at the most elementary level is to perceive the targets’ locations at each time point. The second processing level is to perceive the target objects at different locations and time points as a coherent group of moving targets, while the third level is to continuously allocate attention to the targets while suppressing the non-targets. The various factors mentioned in the prior sections may exert an impact on different levels of processing and thus influence the eye behavior in different ways. Crowding and stimulus size may mainly affect the first level of processing. On the other hand, motion speed, viewpoint change, trajectory repetition, occlusion, and collision may mainly affect the second level. Finally, the number of targets may mainly affect the third level of processing. Thus, eye behavior observed during tracking is likely to reflect a combination of processing at different levels.

The key for the success at the first level of processing is to enhance the resolution of the perceived target locations, so as to maximize the distinction between the target and distracter locations. Small stimulus size may make it difficult to perceive each target location. Thus, the eyes may land closer to targets to perceive the target locations. Moreover, close distance between objects (i.e., crowding) may lead to confusion between the target and distracter locations; in such cases the use of foveal vision is beneficial for resolving the crowding effect. Thus, the eyes are likely to gaze at the areas where targets are crowded by other objects. 

The linking process at the second level can be influenced by various factors. High object speed leads to increased distance between target locations perceived at different time points, while viewpoint-change disrupts the reference frame of the whole scene. In such situations, moving the eyes may further interrupt the processing of object locations, and hence the preferred eye behavior is to maintain the gaze at a central location between the targets. If target trajectories are familiar via repetition, they may facilitate the second-level processing. On the other hand, occlusions and collisions increase the uncertainty of target locations before and after such events. In real-life visual environments, occlusion and collision may hint at the possibility of occurrence of potentially threatening events, so that saccades are automatically programmed toward such events for extracting detailed information with the foveal vision. Whether such rescue saccades are helpful or disruptive to tracking requires further research.

At the third level of processing, cognitive resources are severely limited, so that only a few objects can be simultaneously attended and maintained. In addition, as the number of objects increases, the cognitive resources distributed to each object decreases. Thus, the effect of the number of targets mainly reflects the capacity limitation during MOT. One way to overcome the limitation is to group multiple objects into one single virtual object. Thus, observers are inclined to look at the centroid of multiple targets so as to facilitate the grouping process. 

Eye behavior at the lower processing levels is likely to be bottom-up, automatically induced by the computation of the visual scene, whereas eye behavior serving the high-level processes may be more subject to top-down control. Thus, observers are more likely to become aware of their eye behavior at this level. In sum, to achieve good tracking performance in MOT, the gaze should be optimally anchored to the crowded targets, while monitoring the objects with peripheral vision. Saccades towards targets should be initiated only when the risk of losing track of the targets is greater at the current gaze position than the saccade-related cost [16].

## 3. Eye Behavior during MIT

As described in the Introduction, MIT differs from MOT in that not only the target position but also the target identity needs to be dynamically updated. Arguably, MIT more closely resembles real-life tracking tasks than MOT in the sense that being aware of the target identity is typically relevant to the task at hand. For example, a parent tracking the whereabouts of his/her children on a crowded beach is highly motivated to discriminate his/her own children from the other bathers. 

As MIT has generally been studied to a less extent than MOT, there is only a handful of eye-tracking studies of MIT. They are reviewed next (for their summary, see Table 2). Similarly, to the section on MOT, we first review studies examining where observers look at during MIT, followed by a review of the factors influencing eye movements in MIT and the possible functions of eye movements in MIT.

### 3.1. Where Are Observers Looking at during MIT?

The eye-tracking study of Doran, Hoffman and Scholl [24] is conceived by the authors as a MOT study. However, as they registered observers’ eye movements when they tracked moving lines of different length and orientation, we review it as an MIT study. The targets namely have unique identities defined by their length and orientation. Yet, it isn’t a straightforward MIT study either, because the lines constantly changed their length.

In Experiment 1 of Doran et al. [24], ten observers tracked three target lines out of six for 20 s (Experiment 2 is not reviewed, as the observers were not allowed to move their eyes during tracking). The lines constantly changed length, orientation and velocity with the maximum velocity of 2°/s. After the movement stopped, they were to click with the mouse on the targets. The targets were tracked with 84% accuracy. For the eye movement analysis, four AoIs were determined: centroid, line center, line end, and line location other than center or end. A fixation was assigned to the nearest AoI provided that it was no further than 1° away from it. Line center and end were delineated as AoIs, as they were the two possible probe locations.

Observers spent more time fixating on the targets (~17%) than the centroid (~7%) or the distracters (~5%); the percentages are approximated on the basis of Figure 6 in Doran et al. [24]. Most target fixations were on positions not occupied by the probes (~30%). Doran et al. [24] expected to find evidence for a centroid-looking strategy. They suggest several possibilities for the failure to do so: (a) the stimuli used by them do not have a single locus of attention, (b) the targets often appear in close proximity to each other thus requiring good visual acuity to tell them apart, and/or (c) the probe detection task “may have biased observers’ gaze toward individual objects” [24] (p. 594). In sum, although Doran et al. did not observe any prevalent scanning strategy, the target-switching strategy was more often used than the centroid-looking strategy.

Oksama and Hyönä [20] examined eye behavior during MIT using stimuli that were more comparable to MOT stimuli than the lines used by Doran et al. [24]. In Experiment 2 they registered eye movements during MIT. Observers tracked 2–5 targets among 6 moving objects. The stimuli were line-drawings of real-life objects (e.g., flower, coat). They subtended a maximum visual angle of 1.9 × 1.8°. The same speed conditions (2.6, 6.3, and 10.3°/s) were used as in their Experiment 1 in which eye movements were recorded during MOT (see above). After the movement stopped, all objects were masked, one of the targets was probed, after which the participants were to click on the probed target on a new screen where all 6 objects were displayed.

For the eye fixation analyses, AoIs (3.4° in diameter) were determined for targets, distracters, centroid, and screen center. Oksama and Hyönä [20] found that observers spent the majority of trial time fixating on targets (53%), but very little time on the centroid (1.5%) or distracters (1.7%). The reminder of the time was spent on blank area (25%) outside any AoI. Thus, the prevalent strategy was the target-switching strategy, whereas the centroid-looking strategy frequently found in MOT studies was completely absent.

Li, Oksama and Hyönä [25] used Landolt rings as stimuli in their MIT study. Landolt rings are rings with a gap in some part of the ring. In the study, rings with different identity were created by having the gap appear in different compass orientations. In both experiments of Li et al. [25], observers tracked 3 rings among a total of 8 rings. Their diameter was 1.6°. The rings moved in random directions at the speed of 8.6°/s. Object motion lasted for 3–6 s. After the movement stopped, all objects were masked and one target was probed by presenting it on the screen center. Participants were to click on the probed target or a “Not a target” response option, if (s)he thought it was not a target. Data of 22 participants were included in the analyses of Experiment 1 and those of 25 participants in the analyses of Experiment 2. For the analysis of eye behavior, fixations were assigned to the closest object using 1-degree bins (0–1°, 1–2°, 2–3°, etc.).

Experiment 1 of Li et al. [25] showed that 73% of all fixations landed no farther than 2° from the closest target, whereas only 6% fixations landed close to the centroid. In other words, the target-switching strategy was highly prevalent. Similarly, in Experiment 2, 66% of the fixations were located no further than 2° from a target. The centroid was seldom (6%) looked at.

Li, Oksama and Hyönä [26] investigated how different identities that varied in their visual resolution are tracked. They had participants track faces (high resolution), line drawings (medium resolution) and color disks (low resolution). A pretest demonstrated that these stimuli are identified in the parafovea and periphery with variable success. In the pretest, the stimuli were presented individually 2.5, 5 and 7.5° away from the fixation point. With color discs the identification rate was near ceiling for all these eccentricities, whereas a significant drop as a function of eccentricity was observed for faces. Identification of line drawings was at ceiling for the two nearest eccentricities but dropped in the 7.5° eccentricity.

In Experiment 1, Li et al. [26] examined the tracking of colored, ellipse-shaped facial images (1.7° × 2.3°). Set-size was varied between participants; 20 participants tracked 3 faces among 3 distracter faces, while another 19 participants tracked 4 faces among 4 distracter faces. Faces moved randomly with a speed of 4.5°/s for a period of 4 to 8 s. After the movement stopped, all faces were masked and each target was probed by presenting it on the screen center one by one. The participants were required to click on the probed targets. In Experiment 2 [26], tracking of color discs (2° in diameter) was examined. Each disc appeared in one of nine possible colors. Twenty-one observers tracked 3 target discs among 3 distracter discs; another 19 participants tracked 4 target discs among 4 distracter discs. Experiment 2 was otherwise comparable to Experiment 1. Experiment 3 was comparable to Experiments 1 and 2 apart from the stimuli, which were line drawings (2° × 2°) of common objects. Twenty participants tracked 3 targets among 3 distracters, while another 20 participants tracked 4 targets among 4 distracters. In all three experiments, an AoI of 2.5° around the moving objects was used.

The study of Li et al. [26] established a strong preference for the target-switching strategy; 82% of fixations fell on targets and only 6% on distracters. The target-switching strategy prevailed for all object identities, even though it was somewhat weaker for color discs (73%) than line drawings (85%) or faces (89%). In other words, tracking during MIT appears to be inherently serial and not only limited to high-resolution stimuli.

In sum, the available evidence strongly and consistently suggests that the default eye movement strategy in MIT is the target-switching strategy. The prevalence of the target-switching strategy has been established with different kinds of stimuli.

### 3.2. What Factors Influence Eye Behavior during MIT?

In this section, we review studies that have examined effects of target set-size, target speed and target type on the eye behavior during MIT.

#### 3.2.1. Effects of Set-Size and Speed 

Oksama and Hyönä [20] showed that the number of target visits increased as a function of set-size (2–5 targets), while the fixation time of each visit decreased as a function of set-size. An analogous finding was observed by Li et al. [26] for fixation time when comparing the tracking of 3 versus 4 targets. Oksama and Hyönä [20] also observed a decrease of target visits as a function of speed as well as an interaction between set-size and speed. The interaction suggested that the set-size effect was observable for slow and medium speed but not so much for fast object speed. The number of updated targets (i.e., targets visited with the eyes at least once) showed that target visits closely corresponded with the set-size, indicating that all targets were visited with the eyes at least once. The only exception was set-size 5 combined with fast speed; in this condition participants updated about 4 targets. This finding suggests that with an increase in target speed, observers may not be able to track with their eyes every target, particularly when there are several targets to be tracked. The decrease in target fixation time as a function of set-size indicates that observers move faster their eyes between targets when there are more targets to be tracked.

Oksama and Hyönä [20] also observed that pupil dilated as a function of set-size and speed. Moreover, these effects interacted suggesting that the set-size effect was stronger in the fast than slow speed condition. As pupil size presumably reflects, among other things, attentional effort, these findings suggest that MIT becomes increasingly attentionally demanding with an increase in target set-size and target speed. Finally, the number of blinks was also reduced as a function of set-size and speed. The decrease in blink rate as a function of increase in set-size and speed indicates that observers opt for maximal visual sampling when the task becomes demanding. 

#### 3.2.2. Effects of Target Type

Li et al. [25] conducted two experiments using Landolt rings as stimuli. In Experiment 1, the difficulty of perceiving the identity was varied by manipulating the gap width. The width of the narrow gap was 0.05° and that of the wide gap 0.2°. In Experiment 2, the visibility of the gap was kept constant (the wide gap condition of Experiment 1 was employed), but attentional demands were varied by manipulating the similarity in gap orientation among the targets. The gaps in two rings with a similar gap orientation differed by 20–40°, while the third ring had a gap orientation that differed by more than 80°.

Experiment 1 of Li et al. [25] showed that the probability of fixating on a target at least once during tracking was greater for narrow than wide gaps, which suggests that target fixations are particularly needed for refreshing identity-location bindings for less perceivable identities. In Experiment 2 more fixations were found on attentionally more demanding targets (a similar gap orientation) than less demanding targets (a dissimilar gap orientation). In contrast, fixation duration was shorter on attentionally more demanding than less demanding targets. This trade-off between fixation frequency and duration “may reflect observers’ efforts in keeping the identity of the two similar targets distinct from each other” (p. 620). 

As already mentioned above, Li et al. [26] found that the target-switching strategy prevailed for all tested object identities, color discs, line drawings and faces, although it was somewhat weaker for color discs. Color discs differ from faces and line drawings in that they are more readily perceivable in peripheral vision. 

To sum up, the currently available evidence suggests that the target-switching strategy is the dominant eye movement strategy in MIT regardless of target type. It is even more dominant when a high-resolution representation needs to be constructed for the target identities to tell them apart.

### 3.3. What Functions Do Eye Movements Serve during MIT?

Above, we have reviewed evidence demonstrating the prevalence of the target-switching strategy in MIT. In this section, we review the evidence on the functions of target fixations. The evidence suggests that target fixations serve the purpose of establishing and refreshing identity-location bindings (Section 3.3.1), enhancing the tracking performance (Section 3.3.3) and clustering targets in conflict detection (Section 3.3.4). The present evidence for coupling of the fixation target and the attentional target (Section 3.3.2) suggests that the tightness depends on the type of identity to be tracked.

#### 3.3.1. Establishing and Refreshing Identity-Location Bindings

Li et al. [26] investigated the role of target fixations by employing the gaze-contingent display change paradigm [28,29] to manipulate the availability of the moving objects. This paradigm makes possible to manipulate what is presented in the visual field contingent on where the observer looks at from moment to moment. Four presentation conditions were used: (a) all objects present, (b) only the fixated object visually available (all other objects were replaced with placeholders), (c) all but the fixated object present (once a fixation is initiated on a target, it is masked by a placeholder), and (d) none of the objects present during tracking. In the All-Present condition, observers can utilize both their foveal and peripheral vision for tracking; in the Fovea-Present condition, they can only use foveal vision; in the Periphery-Present condition they can only use peripheral vision; finally, in the None-Present condition tracking is carried out solely with the help of the visuospatial working memory. If only the foveated target is tracked at each moment, the Fovea-Present and All-Present conditions should result in equally good tracking accuracy, whereas the Periphery-Present condition should impair the performance. On the other hand, if multiple identities are tracked simultaneously, the All-Present condition should produce the best performance, followed by the Periphery-Present conditions, as in these conditions multiple identities are simultaneously available.

In Experiment 1 of Li et al. [26], faces were used as stimuli. The results showed that tracking accuracy was practically identical between the Fovea-Present and All-Present conditions (69.5% vs. 69.3%), which produced higher accuracy than the Periphery-Present and None-Present conditions that produced identical accuracy (62.3%). The pattern of results is completely consistent with the view that faces are tracked serially one at a time. Having all targets simultaneously available did not improve the performance from the situation where only the fixated target was available. Moreover, making the foveated target unavailable led to an equally poor performance as when no targets were available.

In Experiment 2 of Li et al. [26], color discs were used as stimuli. With set-size 3 tracking accuracy was near ceiling and it did not differ between the display conditions. With set-size 4, the All-Present and Periphery-Present conditions produced the best performance (86.3% vs. 84.5%) that was significantly better than the accuracy in the Fovea-Present (77.6%) and None-Present (71.7%) conditions. The pattern of results is consistent with the view that color discs are tracked in parallel. The tracking became better with the increase in the number of visually available targets (0, 1, and 3 in the None-Present, Fovea-Present and Periphery-Present conditions, respectively).

In Experiment 3 of Li et al. [26], black-and-white line drawings of common objects were used as stimuli. The All-Present condition produced the best tracking accuracy (89%), followed by the Fovea-Present and Periphery-Present conditions that produced practically equal (86%) accuracy, which was better than for the None-Present condition (82%). The pattern of results suggests that tracking was not completely serial, as seeing targets in the periphery resulted in better performance than seeing no targets. It was not completely parallel either, because seeing multiple targets in the periphery did not result in better performance than seeing just one at fovea. 

Taken together, Li et al. [26] conclude that “the performance accuracy results indicate that the manner of tracking multiple objects varies in the serial-parallel continuum according to the identifiability of the objects” (p. 270). When object identities are readily identifiable in peripheral vision, as is the case with color discs, tracking is parallel, whereas poor peripheral identifiability of object identities, as is the case with facial images, leads to serial tracking. In other words, target fixations are necessary for refreshing identity-location bindings for high-resolution stimuli, but they are not required for tracking low-resolution images. 

Interestingly, observers frequently fixated target locations even when there was nothing to see. This became apparent in the None-Present condition where only the placeholders were visible during tracking and identity tracking needed to be performed by the help of visual-spatial short-term memory. Li et al. [26] found that 75% of dwell time was spent fixating on the target placeholders. The frequency of target visits was not affected by identity type (faces, color discs, line drawings). The frequent visits to placeholders bears resemblance to the “looking-at-nothing phenomenon” [30,31,32]; looking at a location previously occupied by an object may active its memory representation [33]. 

The Periphery-Present condition resembled the None-Present condition in that once a target area was fixated, there was no identity information to be seen in the fovea. Nevertheless, similarly to the None-Present condition, observers made frequent fixations on the placeholders. Moreover, they more frequently visited the placeholders of faces and line drawings than those of color discs. In other words, the above findings reflect observers’ intention of sampling visual information for target identities. 

To sum up, the study of Li et al. [26] demonstrated that in MIT fixations on targets serve the purpose of (a) establishing identity-location bindings for high-resolution stimuli and (b) refreshing identity-location bindings in general. The former function has to do with the need of perceiving the identity of high-resolution stimuli with the foveal vision, while the latter function reflects memory updating. 

#### 3.3.2. Coupling of Attention and Fixation

Three studies have examined the degree to which the attentional target and the fixation target are coupled during MIT. Doran et al. [24] studied tracking of moving lines that constantly changed length, orientation and velocity. Tracking was combined with a secondary task of responding to probes (small grey circles) presented for 213 ms at random intervals either on line center or end. Participants pressed a button as soon as they detected a probe. They were instructed to prioritize tracking over probe detection. Probes were more readily detected when appearing on the line center than end (the so-called concentration effect). This concentration effect was not observed when the fixation happened to be very close (less than 4°) to the probe on the target, probably due to the benefits of high visual acuity for all probes. On the other hand, it was established for farther fixation-probe distances. The concentration effect suggests that attention is more readily concentrated on object centers. Yet, the eye movement data showed that this concentration effect was not coupled with a corresponding concentration of fixations on the object center. Thus, attention and fixation were not tightly coupled.

Li et al. [25] came to a different conclusion regarding the coupling of attention and fixation. They showed that duration of fixations varied as a function of the distance from a target; it was longest when it was no farther than 1° from a target and became shorter as the distance increased up to 4°. The few fixations landing on the centroid did not show such a relationship; instead fixation duration remained stable regardless of its distance to the centroid. The preference for staying fixated on the targets is taken as evidence that these fixations reflect the process of establishing and refreshing the identity-location binding for that target. In Experiment 1, they also found that the probability of fixating on a target at least once during the tracking interval was greater for narrow than wide gaps (Landolt rings were used as stimuli). In Experiment 2, more fixations were found to land on attentionally more demanding targets (a similar gap orientation) than less demanding targets (a dissimilar gap orientation). These results suggest that target fixations are particularly needed for refreshing identity-location bindings for less perceivable and distinguishable identities. Moreover, they suggest a close coupling between the fixation target and the attentional target. 

Finally, the gaze-contingent display change study of Li et al. [26] provided evidence for the view that the coupling of the fixation and attention target depends on the type of identity to be tracked. As reviewed above in more detail, the overall pattern of their results showed that when tracking high-resolution stimuli, the fixation and attention targets are tightly coupled in that observers appear to strongly focus their attention to the fixated target. On the other hand, with low-resolution stimuli tracking is more parallel, meaning that observers simultaneously attend to more than one target. In other words, the attentional target and the fixation target are decoupled. 

To sum up this section, the currently available evidence suggests that the tightness of coupling between attention and fixation during MIT is modified by the type of identity to be tracked. When the targets are readily perceivable in the visual periphery, as is the case with color discs and dot probes, attention and fixation may be decoupled. On the other hand, with high-resolution stimuli requiring foveal vision to be identified attention and fixation are tightly coupled.

#### 3.3.3. Enhancing the Tracking Performance 

Li et al. [25] examined the tracking accuracy as a function of the recency of target fixation. They observed that the recency of target fixation was linearly related to tracking accuracy with most recently fixated targets producing the best tracking accuracy and temporarily more distantly fixated targets having poorer accuracy. This linear trend was slightly steeper for narrow than wide gaps (Landolt rings were used as stimuli). Moreover, the farther away the last fixation was from the probed target, the poorer its tracking accuracy was. This trend was established only for the narrow gap rings. These results show that fixations on targets benefit tracking; on the other hand, when a target is not recently fixated, its identity-location binding is outdated and thus in danger of becoming lost. 

Li et al. [26] replicated the recency effect [25]; targets fixated just before they were probed were associated with better tracking accuracy than previously fixated targets. This held true for faces in all presentation conditions and for line drawings except for the None-Present condition. In other words, the benefit of target fixation was particularly prominent when the targets required high-resolution information to be identified. These findings suggest that eye visits to targets benefit the tracking of high-resolution targets (e.g., faces) but not necessarily low-resolution targets (e.g., color discs). 

#### 3.3.4. Detecting Target Conflicts 

Landry, Sheridan and Yufik [34] registered eye movements in a tracking task where observers’ task was also to detect possible conflicts between targets. The examined tracking task was designed to resemble a task that air traffic controllers are exposed to. “The task was to identify targets predicted to conflict (defined as targets at the same “altitude” that pass within a particular distance on the display of one another)” (p. 93). When a conflict was identified, the participants (14 observers inexperienced in air traffic control) were asked to click on the targets in conflict. As the secondary task, they were to click on new targets appearing on the screen as well as on old targets just before they departed from the screen. Two conflict conditions were created: a demanding (5–7 conflicts) and a less demanding (1–3 conflicts) condition. The number of targets on the screen remained constant (14). Although we review this study as an MIT study, it is not clear how the target identities were marked, as this information is not provided. As the task was to resemble that of air traffic controllers, it is possible that alphabetic call signals were used as target identities. 

Conflict detection among the untrained participants was low, yet the false alarm rate was also low. The eye-tracking data showed that the number of fixations on targets detected as conflicts were not significantly higher than on targets not selected as conflicts. Moreover, there were no more transitions between target pairs detected as conflicts than those not detected. Thus, these results do not support the view that eye fixations would be instrumental in detecting conflicting target trajectories. However, Landry et al. [34] also studied possible clustering of aircraft based on gaze transitions between targets. This was examined by the Virtual Associative Network (VAN) model developed by the authors. VAN represents a unified network of moving objects encompassing the entire visual scene as well as a “dynamic network partitioning into cohesive and externally weakly coupled clusters” (p. 93). Their eye movement analysis revealed that the probability of being within the same cluster was highest (50%) for target pairs correctly detected as being in conflict with each other, and it was lower (30%) for missed conflicts. “This indicates that the ability to detect a conflict may be affected by the ability to group the conflicting targets within a cluster” (p. 99). 

In sum, there is suggestive evidence, based on a single study, that eye movements are used to cluster conflicting targets together when detecting conflicting target trajectories.

### 3.4. Summary of the Eye-Tracking Studies of MIT 

All eye movement studies of MIT demonstrated a preference for using a target-switching strategy. In most studies, the preference was very strong so that up to 80% of the tracking time was spent on fixating targets. 

Constantly fixating targets is not an epiphenomenon in MIT, but target fixations benefit tracking. Most recently fixated targets are associated with better tracking accuracy than more distantly fixated. The target-switching strategy is particularly relevant for high-resolution targets whose identities cannot be perceived peripherally. Target fixations not only serve the purpose of visual sampling and updating of identity information, but they are also in the service of working memory. Even when objects move hidden behind a placeholder, the majority of fixations fall on targets. Fixating the positions of hidden identities boosts their activation in working memory (cf. the looking-at-nothing phenomenon).

## 4. Comparison Eye Behavior in MOT and MIT

Three recent studies [20,27,35] have directly compared eye behavior during MOT and MIT. In this section, we review them one by one.

### 4.1. Oksama and Hyönä (2016)

In Experiment 3 of Oksama and Hyönä [20], performance differences in MOT and MIT were examined by within-participant comparisons. The experimental procedure was identical to that of their Experiment 1 and 2 (see above) with the following exceptions. The MOT stimuli were identical line drawings of a lobster and only half of the trials of Experiment 1 and 2 were presented. MOT and MIT were performed as separate blocks. Data from twelve observers were included in the analyses. 

Tracking accuracy was 90% or better except for set-size 5 in MIT where it went down to approximately 78%. When carrying out the MOT task, observers spent most of their time (48%) looking at a blank space that was neither the centroid nor the screen center, but less time on targets (24%). In contrast, during MIT observers spent most of the time fixating targets (52%) and less time fixating blank space (26%). During MIT, the number of target visits and the number of updated targets increased linearly as a function of set-size, whereas in MOT it remained constant. On average, targets were visited in MIT at the rate of 1.1 Hz, while in MOT the rate was half of that (0.5 Hz). Pupil size increased as a function of set-size, it did so more deeply during MIT than MOT. On the other hand, blink rate decreased as a function of set-size. In sum, participants sampled the dynamic display much more frequently in MIT than in MOT. Moreover, MIT was attentionally more demanding than MOT, as indexed by the pupil size. This was despite the fact that MOT included 10 moving objects (including distracters), whereas MIT included 6 objects. As a result, there was more crowding in MOT and also more direction changes than in MIT. These differences in motion trajectories thus favored MIT over MOT. Nevertheless, MIT turned out to be more demanding than MOT.

Oksama and Hyönä interpret their results to point to two separate tracking systems: “position tracking in the MOT task is achieved by a covert parallel system, whereas identity tracking in the MIT task is achieved by an overt serial system” [20] (p. 407). The position tracking system yields only spatiotemporal information, which is sufficient for MOT but insufficient for MIT. In MIT target identity information needs to be bound with location information, which is a serial process requiring overt attention shifts between targets.

### 4.2. Wu and Wolfe (2018)

Wu and Wolfe [27] were critical of the notion of two parallel tracking mechanisms posited by Oksama and Hyönä [20]. They argue that since Oksama and Hyönä used different stimuli in MOT and MIT, their different results for MOT and MIT may rather reflect stimulus than task differences. In their study, Wu and Wolfe kept the stimuli the same between the two tasks. Both tasks were carried out for hidden stimuli; during the movement phase only the placeholders of objects were visually available. In MOT, participants were required to only keep track of target locations. After the movement stopped, one of the placeholders was probed and the participants responded whether or not the probed circle was a target. In MIT, participants were required to memorize the target identities prior to the movement phase. After the movement stopped, one of the targets was probed, after which a target identity was presented on the screen center and the participants were to respond whether it was the probed target. The targets presented prior to the movement phase (8 s) were 10 cartoon animals (3 × 3° in size), of which observers tracked 3, 4 or 5 that moved with a velocity of 6°/s. MOT and MIT were presented in different blocks. Twelve observers took part in the experiment.

Tracking accuracy was 90% for MOT and 86% for MIT. For the eye movement analyses, four AoIs were delineated: target, distracter, centroid, and everywhere else. The AoI for target, distracter and centroid was 4° in size. Overall, observers spent about 35% of trial time fixating targets, about 35% the area outside targets, distracters and centroid, about 20% fixating the centroid, and about 8% fixating distracters (approximated on the basis of Figure 8 of Wu and Wolfe). Significantly more time was spent in MIT on target fixations and significantly less on centroid fixations than in MOT. Unlike Oksama and Hyönä [20], Wu and Wolfe [27] found no increase in either task in the number of fixations and target visits as a function of set-size (Oksama and Hyönä observed it for MIT but for MOT). The number of updated targets increased in both tasks, but unlike in the Oksama and Hyönä study the increase was not limited to MIT. Finally, a marginal increase was observed in pupil size as a function of set-size. In sum, the qualitative difference in the eye fixation patterns between MOT and MIT observed by Oksama and Hyönä was not replicated by Wu and Wolfe when tracking hidden targets. The main conclusion is that “a serial tracking process is not necessary in MIT since it is still possible to keep track of identities when those identities are hidden during tracking” [27] (p. 459).

### 4.3. Nummenmaa, Oksama, Glerean and Hyönä (2017)

Nummenmaa, Oksama, Glerean and Hyönä [35] studied the neural underpinnings of MOT and MIT in an fMRI investigation. In Experiment 1, they also registered observers’ eye movements. Participants carried out MOT and MIT for 2 and 4 targets among 8 objects that moved for 14–18 s with a variable speed (average speed of 6.3°/s). Tracking accuracy was at ceiling (an average accuracy of 94%) except for set-size 4 in MIT where the accuracy was about 75%. The number of fixations increased as a function of set-size in MIT but in MOT. No AoI analyses were conducted so it is not known where the fixations landed. Saccadic amplitudes were generally longer in MIT than MOT; in MIT they became longer with an increase in set-size but remained constant in MOT. Finally, pupil size increased more steeply during MIT and MOT, as the set-size increased from 2 to 4. In sum, the eye movement results are consistent with those of Oksama and Hyönä [20]. 

The results of brain activation pointed to a shared frontoparietal circuit between MOT and MIT and a unique resource for MIT in dorsolateral prefrontal cortex. The frontoparietal circuit is responsible for the control of attention and eye movements. Although shared between MOT and MIT, it was found to be more strongly activated during MIT than MOT. Dorsolateral prefrontal cortex has an important role in temporarily retaining information particularly in visuospatial working memory —in the case of MIT temporarily storing identity-location bindings.

### 4.4. Summary of the Comparison of MOT and MIT

When MOT and MIT were compared within the same experiment, the target-switching strategy was observed to prevalent in MIT, whereas in MOT participants stayed fixating a blank space or the centroid for longer time than the targets. Thus, these comparisons confirm the results obtained when MOT and MIT were studied separately. A linear increase in target visits was found in MIT as a function of target set-size, but not in MOT. The qualitative changes in eye behavior between MOT and MIT led Oksama and Hyönä [20] posit two distinct tracking mechanisms—a parallel mechanism for position tracking and a serial mechanism for identity tracking. Results on brain activation are compatible with the dual-mechanism view. Wu and Wolfe [27] challenge this view by demonstrating that MOT and MIT produce highly similar eye behavior when tracking hidden objects.

Interestingly, the hidden target tracking employed by Wu and Wolfe [27] is comparable to the None-Present presentation condition of Li et al. [26]. Yet, the results of the two studied differ markedly. Li et al. found that observers looked at the hidden targets 65% of the time, whereas in the Wu and Wolfe study the percentage was much smaller (~35%). A possible explanation for this is that in the Li et al. study hidden target tracking was performed as part of a task where most trials entailed visible objects. Thus, tracking of visibly moving targets may have carried over to hidden target tracking, which was not the case in the Wu and Wolfe study, where only hidden targets were tracked. Li et al. did not include MOT, so a direct comparison to Wu and Wolfe cannot be made. Yet, interestingly Li et al. found no difference in the prevalence of target fixations as a function of identity type. All in all, tracking by memory is an interesting visuospatial ability that deserves further study.

## 5. Future Directions

In the present review, we hope to have demonstrated that eye movements during MOT and MIT are not a mere epiphenomenon, but they play a functional role in tracking of multiple moving objects. This is especially the case in MIT, which more closely resembles tracking in real-life visual environments. In MIT, the default eye behavior is the target-switching strategy (moving the eyes between targets). Although MOT and MIT can be performed with reasonable success without eye movements, keeping the eyes centered on the screen decreases tracking accuracy, particularly for MIT (see e.g., [35]). In MIT, observers move their eyes between targets even when the target identities are occluded, presumably to facilitate refreshing and maintaining identity-location bindings for targets. 

As argued below, future research should focus on MIT, as a more ecologically valid tracking task. Yet, we also think there are interesting issues to be solved with respect to MOT that can ideally be approached using the eye-tracking method. As reviewed above, crowding, possibility for target occlusion and abrupt form changes are likely to trigger a saccade toward “the problem area”. A reasonable assumption is that such rescue saccades are executed so that foveal vision can be brought to bear on keeping track of the targets. Yet, there is evidence suggesting that their execution may actually hinder tracking accuracy. As crowding and occlusion are common phenomena in tracking (be it MOT or MIT), more research should be devoted to examine what role eye behavior plays in preventing disruption by crowding and occlusion. Such results will be relevant to both MOT and MIT.

It is curious that researchers of MOT or MIT motivate their eye-tracking studies by making reference to real-world visual environments (team sports, traffic, crowded areas, etc.) where dynamic tracking of moving objects is an integral part. Yet, practically all the reviewed studies do not make any efforts in mimicking tracking in the real world with three exceptions [10,11,34]. Landry et al. [34] simulated a dynamic visual environment that air traffic controllers deal with. Zelinsky and his group [10,11] mimicked an underwater scene with moving sharks. Thus, it is fair to say that most studies lack ecological validity. Yet, MIT studies are ecologically more valid than MOT studies, as in virtually all real-world situations the to-be-tracked objects have distinct identities. Thus, in the future studies it is preferable to study MIT than MOT, which has not been the case to date.

The lack of ecological validity takes several forms. First, in real-world visual environments motion is quite seldom random, as it has been in the eye movement studies of MOT and MIT reviewed here. Consider, for example, a traffic scene where vehicle motion is heavily constrained by traffic rules. An example of such an approach can be found in the study of Huff, Papermeier and Zacks [36] where the motion of some football players was constrained by the ball motion. Second, in the conducted experiments objects move in a blank space; this is seldom the case in real life. Consider, for example football scene where players motion trajectories are constrained by their position on the field (e.g., near the goal vs. center field). Third, object identity heavily constrains the type of motion an object is capable of performing. For example, human beings are heavily tied to the ground, whereas birds are also equipped for vertical motion. Fourth, the studies have recruited observers inexperienced in tracking of moving objects. As dynamic tracking is a skill likely to improve by practice, it is important to also study expert behavior. Finally, and most importantly, in real-life dynamic environments tracking is not done for its own sake, but object tracking is carried out for the service of the performed task. For example, a football player tracks other players in order to make the decision of his/her next move. Such situations are heavily time-constrained, which means that the player needs to choose what players to track and what players to ignore. In other words, attentional priority is preferentially given to moving objects that are relevant to the performed task. Hence, in future studies MIT should be investigated in task environments where MIT is subsumed into the service of the primary task. This means, among other things, that target designation is not externally given to the observer but is instead determined by the observers themselves.

As reviewed above, eye movements play a functional role particularly in MIT; thus, future MIT studies, preferably along the lines suggested above, should include eye-tracking in their methodological arsenal. Why bother to do that? First, eye movements are closely coupled with overt attention shifts in visually and attentionally demanding tasks such as MIT. Thus, eye-tracking provides useful information about the allocation of attention as it fluctuates over time and space. It is then possible to reveal, for example, what targets are given attentional priority among all targets in a situation when there are multiple targets, of which some are more relevant to the performed task than others. Second, as eye movements are an integral part of MIT, they can be registered without introducing any secondary tasks to measure attentional allocation in MIT. Third, eye-tracking makes possible to study individual differences in the task performance MIT is subsumed into. Taking again an example from football, it would be possible to investigate how expert players track with overt attention other players when preparing to make the decision whom to pass the ball. Their eye behavior may then be compared to more novice players to determine the role of expertise in MIT. Here the role of MIT as a slave mechanism to the primary task is again stressed.

Is it feasible to register eye movements during multiple object tracking in situations mimicking real-world visual environments? Perhaps time is ripe to do so. With recent technological advancements, it has now become possible to register eye movements in virtual reality. Virtual reality itself opens possibilities to simulate and also manipulate visual scenes approximating real-life visual environments. Mobile eye-trackers, on the other hand, make it possible for the researchers to step out “to the wilderness”. A downside of that is that experimentally controlled studies would not be feasible.

## Figures and Tables

**Table 1:** Observed scanning strategies in the eye-tracking studies of multiple object tracking (MOT).

Study	Stimulus Size (°)	Set-Size	Speed (°/s)	Analysis Method	Scanning Strategy
Fehd & Seiffert [8]	2.1	1	15	Shortest distance rule	Target: 96%
Follow-up		3			Centroid: 66%; Target: 9%
		3			Centroid: 42%; Target: 11%
		4			Centroid: 42%; Target 9%
		5			Centroid: 42%; Target 8%
Fehd & Seiffert [9]				AoI: 5° in diameter	
Experiment 1	2.1	4	3, 6, 12, 18, 24		Centroid:~25%; Target: ~10%
Experiment 2	0.06–0.3	4	3, 6, 8, 12, 24		Centroid: 34%; Target: 8%
Experiment 3	1.8	3	12		Centroid: 43%; Target 13%
Zelinsky & Neider [10]	0.5–1.1	1	1.1	Shortest distance rule	Target: 94%
		2			Centroid: 47%; Target: 37%
		3			Centroid: 39%; Target: 42%
		4			Centroid: 24%; Target: 52%
Zelinsky & Todor [11]	0.5–1.1	2–4	Not reported	“Rescue saccade”: landing 1° from a target	Anticipatory rescue saccades were initiated to occluded targets
Huff et al. [12]	1.3–2.2	3		AoI: same size as the objects	
Experiment 1			2		Centroid:~7%; Target: ~10%
			4		Centroid:~7.5%; Target:~8.5%
			6		Centroid:~10%; Target:~8%
Experiment 2			4		Centroid:~11%; Target:~8%
			10		Centroid:~12.5%; Target:~5.5%
Vater et al. [13]	1	4	6, 9, 12	AoI: 5° in diameter	Centroid: 30%; Target: 11%
Vater et al. [14]	1	4	6	Gaze-vector distances	Gaze was closer to centroid than to target regardless of target changes
Vater et al. [15]	1	4	6	Relative gaze distance to the targets	Gaze was closer to crowded than uncrowded targets. Anticipatory saccades were initiated to targets colliding with border.
Lukavský [16]	1	4	5	AoI: 1° for centroid, 2° for objects; Normalized scanpath saliency	Centroid: 7.7%; Target: 12.6%; Anticrowding point: 12.2%; Target eccentricity minimizing point: 9%
Dechterenko & Lukavsky [17]	1	4	Adaptive	Normalized scanpath saliency	The model accounting for the crowding effect yielded the best performance.
Lukavský & Děchtěrenko [18]	1	4	5	Local maximum of similarity	Gaze position lagged by approximately 110 ms behind the scene content.
Děchtěrenko, Lukavský, & Holmqvist [19]	1	4	5	Correlation distance	Scan patterns in flipped trials differed only slightly from those of the original trials.
Oksama & Hyönä [20]					
Experiment 1	2.1	2–5	2.6, 6.3, 10.3	AoI: 3.4°	Blank area: 48%; Target: 21%; Centroid: 7%; Distracter: 4%
Experiment 3	2.1	2–5	2.6, 6.3, 10.3	AoI: 3.4°	Blank area: 48%; Target: 24%; Centroid: 7%; Distracter: 5%

**Table 2:** Observed scanning strategies in eye-tracking studies of multiple identity tracking (MIT).

Study	Stimuli	Set-Size	Speed (deg/s)	Analysis Method	Scanning Strategy
Doran, Hoffman & Scholl [24]	lines that varied in size	3	2	AoI: 1 deg	Target: ~17%; Centroid: ~7%; Distracter: ~5%
Oksama & Hyönä [20]					
Experiment 2	line drawings 1.9 × 1.8 deg	2–5	2.6, 6.3, 10.3	AoI: 3.4 deg	Target: 53%; Blank area: 25%; Centroid: 2%; Distracter: 2%
Experiment 3	line drawings 1.9 × 1.8 deg	2–5	2.6, 6.3, 10.3	AoI: 3.4 deg	Target: 52%; Blank area: 24%; Centroid: 2%; Distracter: 2%
Li, Oksama & Hyönä [25]	Landolt rings, 1.6 deg	3	8.6	AoI: 2 deg	
Experiment 1					Target: 73%; Centroid: 6%
Experiment 2					Target: 66%; Centroid: 6%
Li, Oksama & Hyönä [26]	faces (1.7–2.3 deg), color discs (2 deg), line drawings (2 × 2 deg)	3,4	4.5	AoI: 2.5 deg	
All-Present					Target: 82%; Distracter: 6%
None-Present					Target: 76%
Wu & Wolfe [27]	hidden animals (3 × 3 deg)	3–5	6	AoI: 4 deg	Target: ~35%; Blank area: ~35%; Centroid: ~20%; Distracter: ~8%
